# TEAD1 Prevents Necroptosis and Inflammation in Cisplatin-Induced Acute Kidney Injury Through Maintaining Mitochondrial Function

**DOI:** 10.7150/ijbs.104335

**Published:** 2025-01-01

**Authors:** Melanie Tran, Baihai Jiao, Hao Du, Dong Zhou, Vijay Yechoor, Yanlin Wang

**Affiliations:** 1 Division of Nephrology, Department of Medicine, University of Connecticut School of Medicine, Farmington, CT, USA.; 2 Department of Cell Biology, University of Connecticut School of Medicine, Farmington, CT, USA.; 3 Department of Medicine, University of Pittsburg, Pittsburg, PA, USA.; 4 Renal Section, Veterans Affairs Connecticut Healthcare System, West Haven, CT, USA.

**Keywords:** acute kidney injury, mitochondria, necroptosis, inflammation

## Abstract

Cisplatin is widely used for the treatment of solid tumors and its antitumor effects are well established. However, a known complication of cisplatin administration is acute kidney injury (AKI). In this study, we examined the role of TEA domain family member 1 (TEAD1) in the pathogenesis of cisplatin-induced AKI. TEAD1 expression was upregulated in tubular epithelial cells of kidneys with cisplatin-induced AKI. TEAD1 floxed mice (TEAD1^CON^) mice treated with cisplatin developed tubular cell damage and impaired kidney function. In contrast, proximal tubule specific TEAD1 knockout (TEAD1^PKO^) mice treated with cisplatin had enhanced tubular cell damage and kidney dysfunction. Additionally, TEAD1^PKO^ mice treated with cisplatin had augmented necroptotic cell death and inflammatory response compared to TEAD1^CON^ mice with cisplatin. Knockdown of TEAD1 in mouse tubular epithelial cells showed increased intracellular ROS levels, reduced ATP production and impaired mitochondrial bioenergetics compared to control cells treated with cisplatin. Mechanistically, TEAD1 interacts with peroxisomal proliferator-γ coactivator-1α (PGC-1α), a master regulator of mitochondrial biogenesis, to promote mitochondrial function. Taken together, our results indicate TEAD1 plays an important role in the pathogenesis of cisplatin-induced AKI through regulation of necroptosis and inflammation, which is associated with mitochondrial metabolism. Therefore, TEAD1 may represent a novel therapeutic target for cisplatin-induced AKI.

## Introduction

*Cis*-diamminedichloroplatinum (cisplatin) is an inorganic platinum-based chemotherapeutic agent that is widely used for the treatment of solid tumors 1-3. Although cisplatin is an effective chemotherapeutic drug for cancer treatment, its clinical application is limited due to its side effects, such as toxic accumulation in tubular epithelial cells resulting in nephrotoxicity 4, 5. Approximately one third of patients develop renal dysfunction following treatment with cisplatin, and therefore cisplatin-induced acute kidney injury (AKI) is considered one of the major side effects of cisplatin therapy. The pathophysiology of cisplatin nephrotoxicity has been studied for many decades. However, only recently has research been directed towards comprehending the cellular and molecular mechanisms underlying cisplatin nephrotoxicity. These mechanisms include cell death, generation of reactive oxygen species (ROS), mitochondrial dysfunction, DNA damage, and inflammation 6-8. Thus, there remains an urgent need to find an effective therapy to prevent the complications of cisplatin-induced AKI.

The TEA domain family (TEAD) of transcription factors are one of the major downstream effectors of the Hippo signaling pathway. TEADs are evolutionary conserved, ubiquitously expressed in most tissues, and play important roles in the regulation of tissue homeostasis 9-11. Of the four TEAD factors (TEAD1-4), TEAD1 has the highest expression in the kidney. Although TEAD factors bind to a consensus DNA sequence 5'-CATTCC-3' called the “MCAT (muscle CAT) element,” they do not have a transactivation or transrepression domain and require co-factors like yes-associated protein (YAP) and PDZ-binding motif (TAZ) for transcriptional regulation 8, 9, 12. TEAD1 has previously been shown to regulate cellular proliferation, oncogenesis, differentiation, and control of organ size 13, 14 and is involved in a variety of diseases including cardiovascular disease, polycystic kidney disease, liver regeneration, and tumors 13-18. A recent study showed that deletion of TEAD1 in murine cardiomyocytes activated the necroptotic pathway which was associated with TEAD1 regulating expression of nuclear DNA-encoded mitochondrial genes required for ATP synthesis 19-21. Moreover, YAP-TEAD1 signaling could also induce mitochondrial biogenesis in endothelial cells to stimulate angiogenesis through peroxisomal proliferator-γ coactivator-1α (PGC1α) 22, 23. However, the function of TEAD1 in acute kidney injury is largely unexplored.

In this study, we investigated the pathophysiological role of TEAD1 in cisplatin-induced AKI, focusing on its role in mediating cisplatin-induced effects on mitochondrial function. Our results demonstrate that deletion of TEAD1 in proximal tubular epithelial cells of mice worsens cisplatin-induced kidney dysfunction and tubular cell injury, necroptosis, and inflammation associated with impaired mitochondrial function. These findings demonstrate that TEAD1 regulates cisplatin-induced AKI and suggest that increasing TEAD1 may be a potential approach to alleviate AKI following administration of cisplatin.

## Methods

### Animals

All animal procedures were conducted in accordance with the National Institutes of Health (NIH) Guide for the Care and Use of Laboratory Animals. All experiments were approved by the Institutional Animal Care and Use Committee of the University of Connecticut Health Center. TEAD1^flox/flox^ mice 20 on a C57BL/6J background were mated with mice that express the Cre recombinase under the control of the phosphoenolpyruvate carboxykinase (PEPCK) promoter 24 to generate mice with proximal-tubule-specific deletion of TEAD1 (Supplementary Figure S1A). Genotyping was confirmed by PCR. Male mice were used for all experimental procedures due to variations in the female estrus cycle which can influence kidney damage. Age-matched (10-12 weeks of age) male mice were maintained on a 12 h light-dark cycle in a temperature (22ºC) and humidity controlled (45-55%) environment with ad libitum access to food and water. Proximal-tubule-specific TEAD1 knockout mice (TEAD1^PKO^) and their littermates, TEAD1^flox/flox^ mice (TEAD1^CON^), were randomly assigned a treatment group and were administered a single intraperitoneal dose of cisplatin (20 mg/kg) or vehicle (0.9% saline). Animals were euthanized at 72 h after cisplatin or vehicle administration and blood and kidneys were collected for further analysis. Mice were monitored following administration of cisplatin for adverse effects and were euthanized if mice did not look healthy. Unless otherwise specified, over 6 mice per group were used in each experiment.

### Reagents and antibodies

Cisplatin and anti-NGAL antibody (1:1000 dilution) were obtained from R&D Systems (Minneapolis, MN, USA). Anti-GAPDH (1:5000 dilution) antibody was purchased from EMD Millipore (Burlington, MA, USA). Anti-TEAD1 (1:1000 dilution for immunoblotting, 1:100 dilution for immunostaining), anti-phospho-RIP1 (Ser166, 1:1000 dilution), anti-RIP (1:1000 dilution), anti-phospho-RIP3 (Thr231/Thr232, 1:1000 dilution), anti-RIP3 (1:1000 dilution), anti-phospho-MLKL (Ser345, 1:1000 dilution), anti-MLKL (1:1000 dilution), anti-caspase-3 (1:1000 dilution for immunoblotting), anti-cleaved caspase-3 (1:1000 dilution for immunoblotting, 1:100 dilution for immunostaining), anti-F4/80 (1:100 for immunostaining) and anti-TOM20 (1:1000 dilution) antibodies were purchased from Cell Signaling Technology (Danvers, MA, USA). Anti-PGC1α (1:1000 dilution) and anti-TEAD1 (1:1000 dilution for immunoblotting) antibodies were purchased from Proteintech (Rosemont, IL, USA) and BD Biosciences (Bedford, MA, USA), respectively. Anti-DRP1 (1:500 dilution) and anti-BAX (1:1000 dilution) were obtained from Santa Cruz Biotechnology (Dallas, TX, USA). Donkey anti-rabbit IgG (HRP, A-11034, 1:5000 dilution) and donkey anti-mouse IgG (HRP, A-11005, 1;5000 dilution) were purchased from Thermo Fisher Scientific (Waltham, MA, USA). The plasmids, pLKO.1-vector and pLKO-shTEAD1, were kindly gifted by Dr. Jiliang Zhou (Augusta University, Augusta, GE, USA).

### Generation of stable cell line

Mouse renal tubular epithelial (TCMK-1) and human HEK293T cells were obtained from the American Type Culture Collection (Manassas, VA, USA) and cultured as recommended in Dulbecco's modified Eagle's medium (DMEM) containing 10% fetal bovine serum and 1% penicillin and streptomycin, respectively. To knock down TEAD1, HEK293T cells were transfected with shRNA targeting TEAD1 (shTEAD1) or pLKO.1 (shCON) using Lipofectamine 2000 Reagent to produce lentiviral particle solutions. The TCMK-1 cells were seeded onto 6-well plates and then incubated with TEAD1 shRNA or CON shRNA lentiviral particle solution for 24 hours followed by selection with puromycin (2 μg/ml) for one week. The knockdown of TEAD1 was confirmed by Western blot analysis.

### Western blotting and co-immunoprecipitation

Total proteins were extracted using RIPA buffer containing a cocktail of protease and phosphatase inhibitors (Thermo Fisher Scientific). Equal amounts of protein were separated onto SDS-polyacrylamide gels and then transferred onto nitrocellulose membranes. After blocking in 5% milk for 1 h, membranes were incubated in specific primary antibodies overnight at 4°C, followed by incubation with appropriate HRP-conjugated secondary antibodies for 1 hour. Proteins of interest were analyzed using a scanner, and signal intensities were quantified using ImageJ software (National Institutes of Health). For co-immunoprecipitation assay, TCMK-1 cells were lysed in NP-40 lysis buffer (Thermo Fisher Scientific) containing a cocktail of protease and phosphatase inhibitors. 5% of lysates were kept as whole cell lysates. The remaining lysates were pre-cleared with protein A/G Plus-agarose (Santa Cruz Biotechnology) for 1 h, then IP lysates were incubated with rabbit IgG or rabbit anti-TEAD1 antibody overnight at 4°C. The protein-antibody complex was precipitated with protein A/G Plus-agarose for 2 h and eluted with 1x reducing sample buffer.

### Immunohistochemistry

Kidney sections were deparaffinized and rehydrated in concentrations of ethanol. Antigen retrieval was performed with antigen unmasking solution (Vector Laboratories, Burlingame, CA, USA) followed by blocking of endogenous peroxidase activity quenched with 3% H_2_O_2_. Sections were blocked with 5% normal serum for 1 h, and then kidney sections were incubated with specific primary antibodies overnight at 4°C in a humidified chamber. The following day, sections were washed with PBS, and then incubated with the corresponding secondary antibodies followed by ABC solution sequentially using the ABC kit (Vector Laboratories). Immunoreactivities were visualized with DAB solution (Vector Laboratories), and kidney sections were counterstained with hematoxylin. Images were visualized on a Nikon microscope and quantification was performed in a blinded manner on five randomly selected fields from each kidney section.

### Quantitative real-time RT-PCR

TRIzol reagent was used to extract RNA from kidney tissues (Thermo Fisher Scientific). Aliquots (1 μg) of total RNA were reverse transcribed using SuperScript II reverse transcriptase (Bio-Rad, Hercules, CA, USA) and real-time RT-PCR was performed using SYBR Green Supermix reagent (Bio-Rad) according to the manufacturer's instructions. The comparative threshold cycle (C_T_) method (ΔΔC_T_) was used to quantify gene expression, and relative quantification was calculated as 2-ΔΔCT. Expression levels of target genes were normalized to the GAPDH level in each sample 25. The primers utilized are listed in Table S1.

### Assessment of kidney function

Serum creatinine and BUN levels were measured using commercially available kits (BioAssay Systems, Hayward, CA, USA) according to the manufacturer's instructions as previously described 26.

### Evaluation of kidney morphology

Kidney tissues were fixed in 10% buffered formalin, embedded in paraffin wax, and cut at 4 μm thickness. Kidney sections were deparaffinized, dehydrated, and then stained with hematoxylin and eosin. Tissue damage was examined in a blinded manner and scored on a five-point scale according to the percentage of observed damaged tubules: 0 (<10%; absent), 1 (<25% damage; mild) 2 (26-50%; mild), 3 (51-75%; moderate) and 4 (>75%; severe) 27, 28. Five randomly selected fields from each group were scored, and the average score was calculated as the tubulointerstitial injury score 27, 28.

### TUNEL assay

Apoptotic cell death was evaluated in paraffin-embedded kidney sections using a fluorometric terminal deoxynucleotidyl transferase-mediated digoxigenin-deoxyuridine nick-end labeling (TUNEL) assay kit (Promega, Wisconsin, WI, USA) according to the manufacturers' instructions. Sections were scanned using a microscope equipped with a digital camera (Nikon Instruments), and quantitative evaluation was performed using NIS-Elements Br 3.0 software (Nikon Instruments). The number of TUNEL-positive cells per high-power field were counted and analyzed in a blinded manner 26, 29.

### ATP measurement

ATP levels were measured in TCMK-1 cells using an ATP assay kit according to the manufacturer's instructions (Abcam). Briefly, cells were harvested in ATP assay buffer, and samples were run in duplicate in ATP reaction mix containing assay buffer, ATP probe, ATP converter and developer mix. After incubation at room temperature for 20 min, the plate was measured calorimetrically at OD 570nm and levels of ATP in each sample was calculated and normalized to the protein concentration.

### Mitochondrial morphology and detection of mitochondrial ROS

Mouse TCMK-1 cells were seeded onto black 96 well plates. After overnight serum starvation, cells were treated with cisplatin (10 μM) or vehicle (PBS) for 24 h. For detection of intracellular ROS, cells were incubated with 10 μM 2′,7′-dichlorodihydrofluorescein diacetate (DCFH-DA, Abcam, Waltham, MA, USA) at 37°C for 20 minutes. The conversion of DCFH-DA to the fluorescent product DCF was measured fluorometrically at Ex/Em = 485/535 nm. For MitoSOX (Thermo Fisher Scientific) determination, plated cells were washed with PBS and then incubated with 10 mM MitoSOX for 15 minutes in the dark, and the fluorescent intensity was measured on the plate reader at Ex/Em = 490/610 nm. To label mitochondria, TCMK-1 cells were seeded and grown onto glass cover slips. Cells were serum starved overnight followed by treatment with 10 µM cisplatin for 24 h. The following day, cells were labelled with 50 nM MitoTracker Red CMXRos (Thermo Fisher Scientific) in DMEM for 30 min at 37°C. Cells were washed with PBS and mounted onto glass slides using mounting medium containing DAPI (Vector Laboratories).

### Seahorse analysis

Approximately 1 x 10^4^ cells/ml was seeded onto a Seahorse XFe96 plate in XF DMEM assay medium supplemented with 1mM XF glucose, 1mM XF glutamine, and 1mM XF pyruvate. After overnight serum starvation, cells were treated with 10 μM cisplatin for 24 h and then cellular mitochondrial function was measured using a Seahorse XF96 Extracellular Flux Analyzer and a Seahorse XF Cell Mito Stress Kit (Agilent Technologies, CA, USA). After replacing the culture medium with seahorse buffer, oligomycin (1.5 mol/L), FCCP (0.5 mol/L), and rotenone/antimycin A (0.5 mol/L) were automatically injected into each well of the plate. Oxygen consumption rate (OCR) was normalized to cell lysate protein content. Analysis was performed using the Agilent software provided by the manufacturer.

### Statistical analysis

Data are presented as mean ± standard error of the mean (SEM) and were analyzed using GraphPad Prism software (Version 8.0). Analysis of two groups was performed using a two-tailed student's *t*-test. Multiple-group comparisons were performed by ANOVA followed by the Bonferroni procedure for comparison of means. *P* values of <0.05 were considered statistically significant.

## Results

### TEAD1 expression is upregulated in tubular epithelial cells of mice with cisplatin-induced AKI

**Figure 1:**
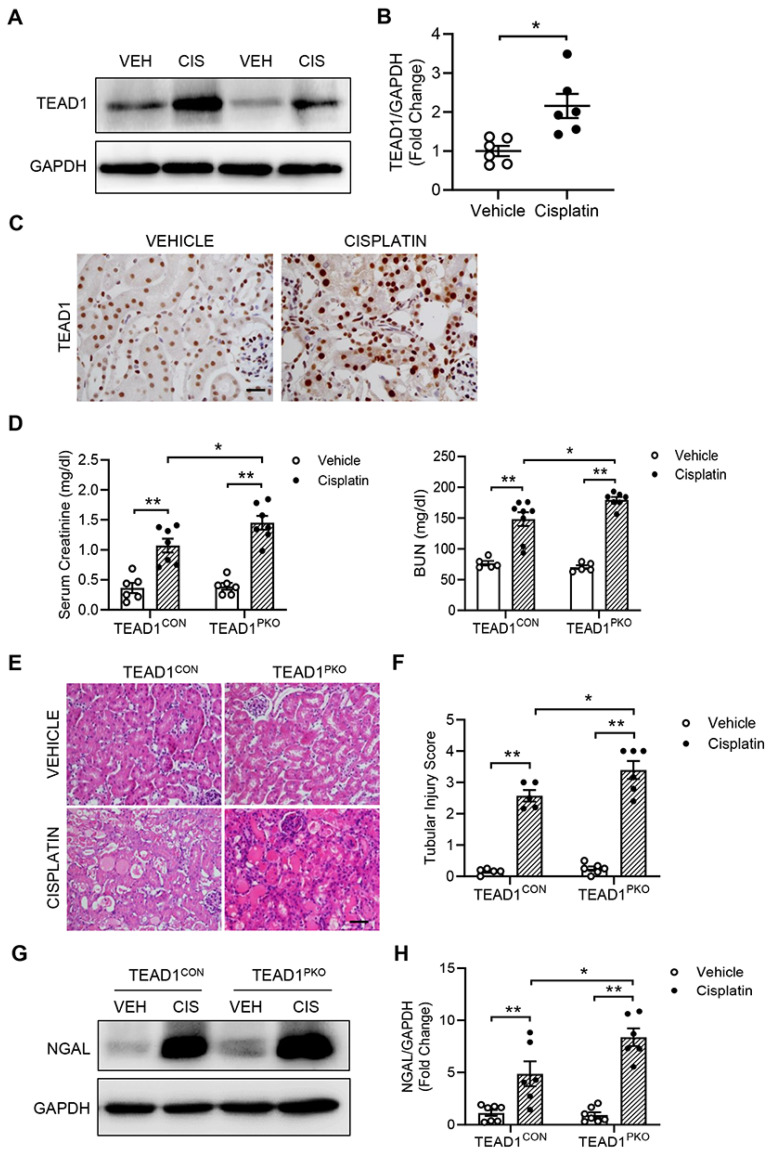
**TEAD1 deficiency induces tubular cell damage and kidney dysfunction.** (A) Representative Western blots show TEAD1 protein levels in kidneys of wildtype (WT) mice at 72 h after cisplatin or saline administration (B) Quantitative analysis of TEAD1 protein expression in kidneys of WT mice. ** *P* < 0.01, n = 6 per group (C) Representative photomicrographs of kidney sections stained for TEAD1 (brown) and counterstained with hematoxylin (blue). Scale bar: 50 μM (D) Serum creatinine and BUN levels at 72 h after cisplatin or saline administration. * *P* < 0.05 and ** *P* < 0.01, n = 6-8 per group (E) Representative photomicrographs of hematoxylin and eosin (H&E) staining in kidneys of TEAD1^CON^ and TEAD1^PKO^ mice at 72 h after cisplatin or saline administration. Scale bar: 50 μM (F) Quantitative assessment of tubular damage in mice at 72 h after cisplatin or saline administration. * *P* < 0.05 and ** *P* < 0.01, n = 6 per group (G) Representative Western blots show NGAL protein expression in kidneys of TEAD1^CON^ and TEAD1^PKO^ mice at 72 h after cisplatin or saline administration (H) Quantitative analysis of NGAL protein expression in kidneys of mice at 72 h after cisplatin or saline administration. * *P* < 0.05 and ** *P* < 0.01, n = 6-7 per group.

To investigate the role of TEAD1 in cisplatin-induced AKI, we examined the protein expression of TEAD1 in kidneys. Western blot analysis showed that TEAD1 expression was significantly upregulated in the kidneys of mice with cisplatin-induced AKI compared with mice treated with vehicle (Figure 1A-B). To determine the localization of TEAD1, immunohistochemistry was performed in kidneys with vehicle or cisplatin treatment. Immunohistochemical staining showed that TEAD1 was predominantly expressed in the tubular epithelial cells of the kidney, with some positive TEAD1 staining observed in the tubulointerstitial area of the kidney (Figure 1C). These results indicate that cisplatin treatment induced TEAD1 expression in the tubular epithelial cells of the kidneys in mice.

### TEAD1 deficiency promotes tubular cell damage and kidney dysfunction

Cisplatin nephrotoxicity occurs mainly in the tubular epithelial cells of the kidney. Therefore, we crossed TEAD1^flox/flox^ (TEAD1^CON^) mice with PEPCKCre^+/-^ mice to generate proximal-tubule-specific TEAD1 knockout mice (TEAD1^PKO^) and examined the role of proximal tubule TEAD1 in the pathogenesis of AKI induced by cisplatin administration (Supplementary Figure S1A).

Immunohistochemical staining for TEAD1 confirmed deletion of TEAD1 in kidney proximal tubules of TEAD1^PKO^ mice (Supplementary Figure S1B). On day 3 after the AKI induction, cisplatin administration resulted in elevated serum creatinine and BUN levels in TEAD1^CON^ mice which was further elevated in TEAD1^PKO^ mice (Figure 1D). Moreover, H&E staining showed that kidneys from cisplatin group demonstrated typical features of tubular injury, including tubular dilatation, loss of brush border, cytoplasmic vacuoles, cast formation, and inflammatory cell infiltration compared with Vehicle group (Figure 1E). In TEAD1^PKO^ mice, cisplatin induced a greater tubular injury score compared to TEAD1^CON^ mice (Figure 1E-F). Furthermore, neutrophil gelatinase-associated lipocalin (NGAL), an early biomarker of renal tubular damage, was significantly upregulated in both TEAD1^CON^ and TEAD1^PKO^ mice following cisplatin treatment, but higher in TEAD1^PKO^ mice (Figure 1G-H). These data indicate that genetic deletion of TEAD1 promotes tubular cell damage and kidney dysfunction in response to cisplatin administration.

### TEAD1 deficiency is independent of tubular apoptosis in cisplatin-induced AKI

**Figure 2:**
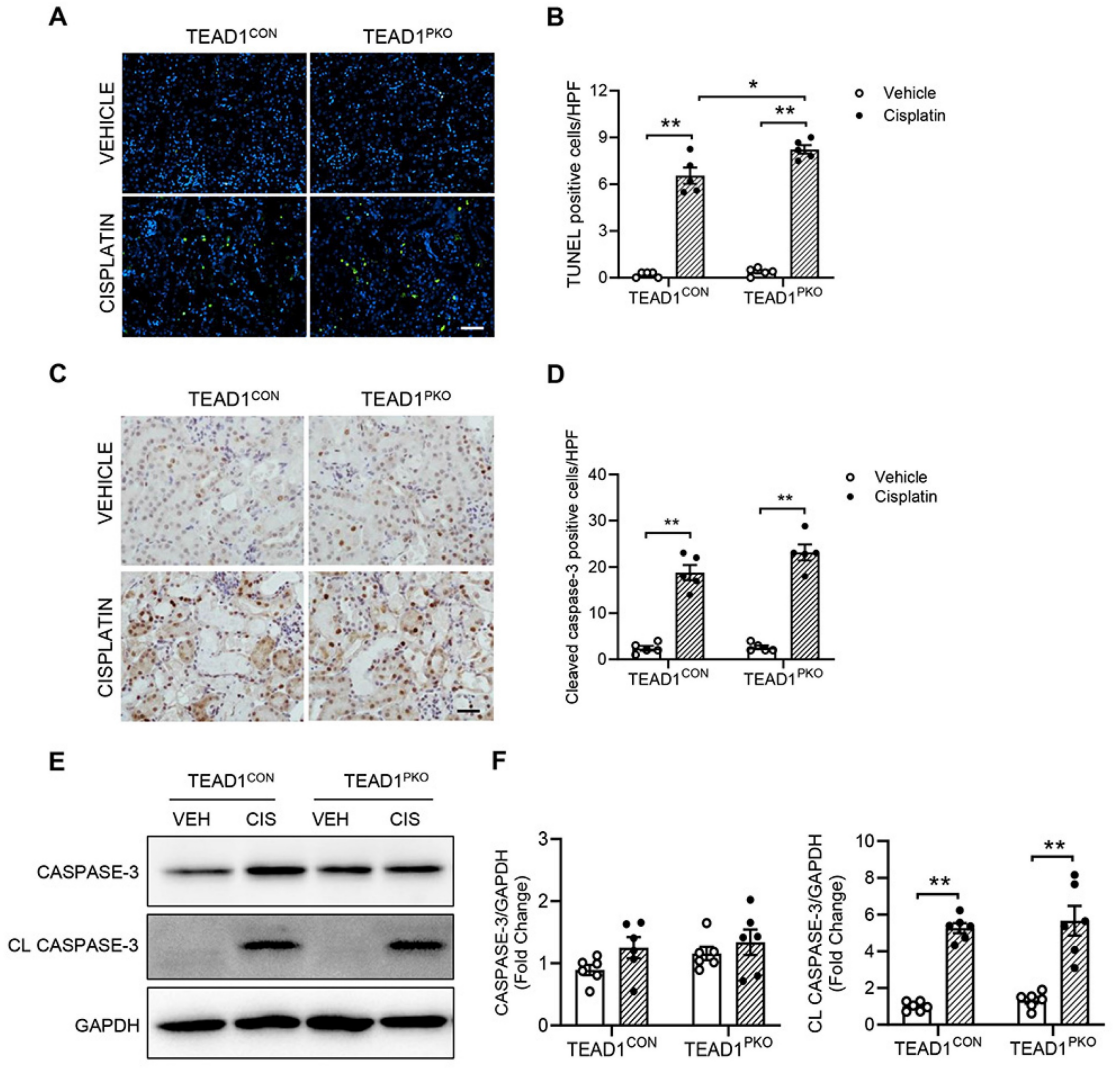
**TEAD1 deficiency is independent of tubular apoptosis in cisplatin-induced AKI.** (A) Representative photomicrographs of kidney sections immunostained for apoptotic cells using terminal deoxynucleotidyl transferase dUTP nick end labeling (TUNEL) in TEAD1^CON^ and TEAD1^PKO^ mice at 72 h after cisplatin or saline administration. Scale bar: 100 µM (B) Quantitative analysis of TUNEL positive cells in kidneys of mice at 72 h after cisplatin or saline administration. * *P* < 0.05 and ** *P* < 0.01, n = 5 per group (C) Representative photomicrographs of kidney sections immunostained for cleaved caspase-3 (brown) and counterstained with hematoxylin in TEAD1^CON^ and TEAD1^PKO^ mice at 72 h after cisplatin or saline administration. Scale bar: 50 μM (D) Quantitative analysis of cleaved caspase-3 positive cells in kidneys of mice at 72 h after cisplatin or saline administration. * *P* < 0.01 and ** *P* < 0.01, n = 5 per group (E) Representative Western blots show caspase-3 and cleaved caspase-3 protein expression in kidneys of TEAD1^CON^ and TEAD1^PKO^ mice at 72 h after cisplatin or saline administration (F) Quantitative analysis of caspase-3 and cleaved caspase-3 protein expression in mice at 72 h after cisplatin or saline administration. * *P* < 0.05 and ** *P* < 0.01, n = 6 per group.

A major feature of cisplatin-induced AKI is apoptosis of kidney tubular cells. To determine whether TEAD1 deficiency affected cisplatin-induced apoptosis in kidneys, we performed a TUNEL assay. In both TEAD1^PKO^ and TEAD1^CON^ kidneys, the number of TUNEL-positive cells was upregulated following cisplatin treatment compared to vehicle groups, but higher in TEAD1^PKO^ kidneys compared to TEAD1^CON^ kidneys following cisplatin treatment (Figure 2A-B). Next, we immunostained kidney sections for cleaved caspase-3 and found that in mice treated with cisplatin, the number of cleaved caspase-3 positive cells was similar between TEAD1^CON^ and TEAD1^PKO^ mice (Figure 2C-D). Accordingly, western blot analysis showed that cleaved caspase-3 was similarly increased in kidneys of TEAD1^CON^ and TEAD1^PKO^ mice after cisplatin treatment (Figure 2E-F). Taken together, these results demonstrate that augmentation of cisplatin-induced AKI by deletion of *TEAD1 in mice* is independent of tubular cell apoptosis.

### TEAD1 deficiency promotes necroptosis-induced cell death in cisplatin-induced AKI

**Figure 3:**
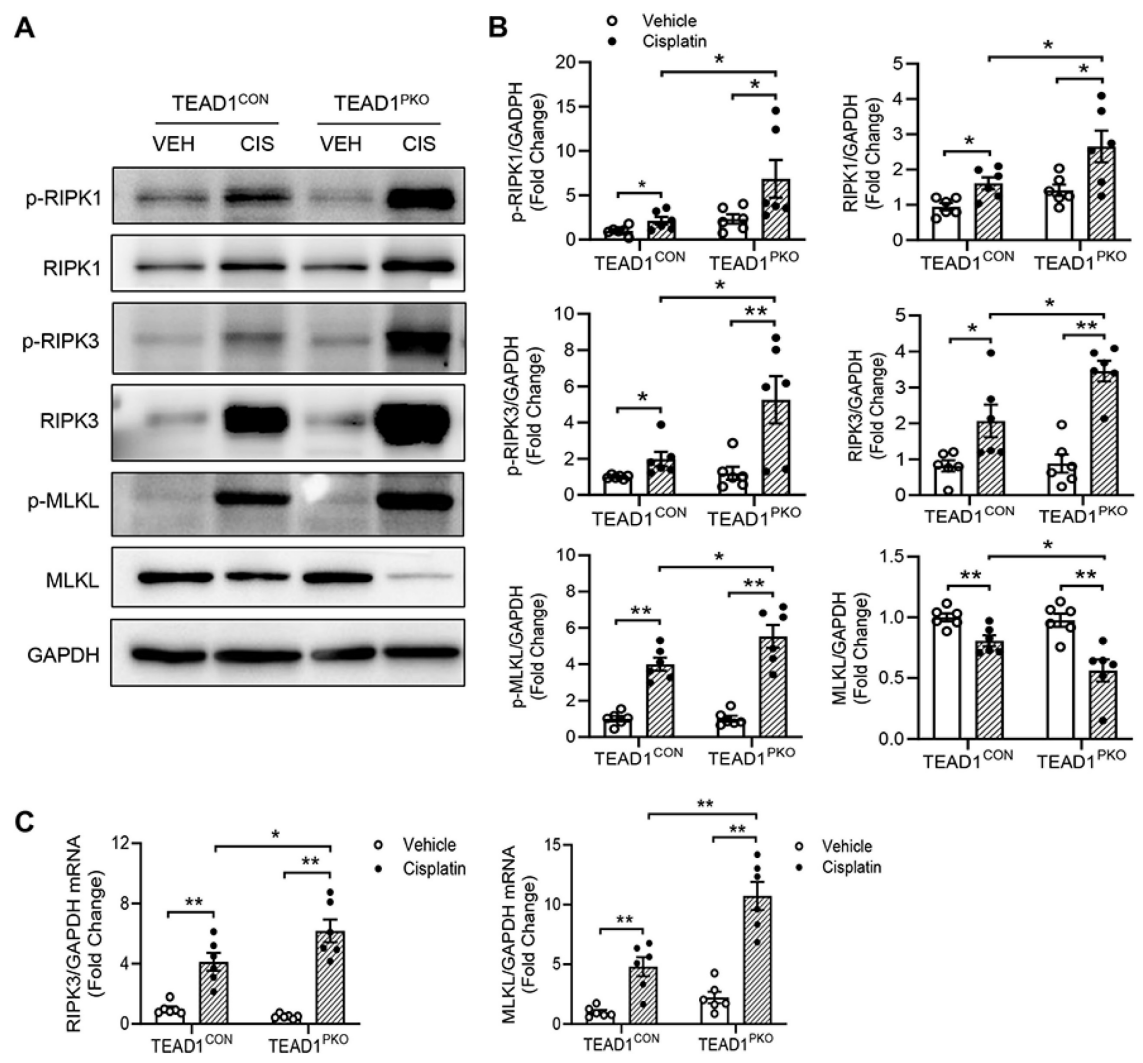
** TEAD1 deficiency promotes necroptosis-induced cell death in cisplatin-induced AKI.** (A) Representative Western blots show phosphorylated RIP1, RIP1, phosphorylated RIP3, RIP3, phosphorylated MLKL and MLKL protein expression in kidneys of TEAD1^CON^ and TEAD1^PKO^ mice at 72 h after cisplatin or saline administration (B) Quantitative analysis of phosphorylated RIP1, RIP1, phosphorylated RIP3, RIP3, phosphorylated MLKL and MLKL protein expression in kidneys of mice at 72 h after cisplatin or saline administration. * *P* < 0.05 and ** *P* < 0.01, n = 6 per group (C) Quantitative analysis of RIP3 and MLKL mRNA expression in kidneys of TEAD1^CON^ and TEAD1^PKO^ mice at 72 h after cisplatin or saline administration. * *P* < 0.05 and ** *P* < 0.01, n = 6 per group.

Necroptosis, a type of programmed cell death, has been reported to be involved in cisplatin-induced AKI 30, 31. The protein expression of the core components of the necroptotic pathway including phosphorylated MLKL, RIP1, and RIP3 were examined in the kidneys of mice with cisplatin administration. Western blot analysis showed that TEAD1^CON^ mice treated with cisplatin had increased expression of phosphorylated RIP1, RIP3 and MLKL compared with vehicle-treated TEAD1^CON^ mice (Figure 3A-B). This expression was further upregulated in the kidneys of TEAD1^PKO^ mice treated with cisplatin (Figure 3A-B). Similarly, the mRNA expression of necroptotic genes, including RIP3 and MLKL, increased in TEAD1^CON^ mice following treatment with cisplatin and more so in TEAD1^PKO^ mice (Figure 3C). In TCMK-1 cells treated with 10 µM cisplatin for 24 h, knockdown of TEAD1 also showed increased RIP1 and MLKL phosphorylation while RIP3 phosphorylation remained unchanged (Supplementary Figure S2A-B). Our results indicate that genetic deletion of TEAD1 in mice promotes necroptosis-induced cell death in cisplatin-induced AKI.

### TEAD1 deficiency upregulates inflammatory response in cisplatin-induced AKI

**Figure 4:**
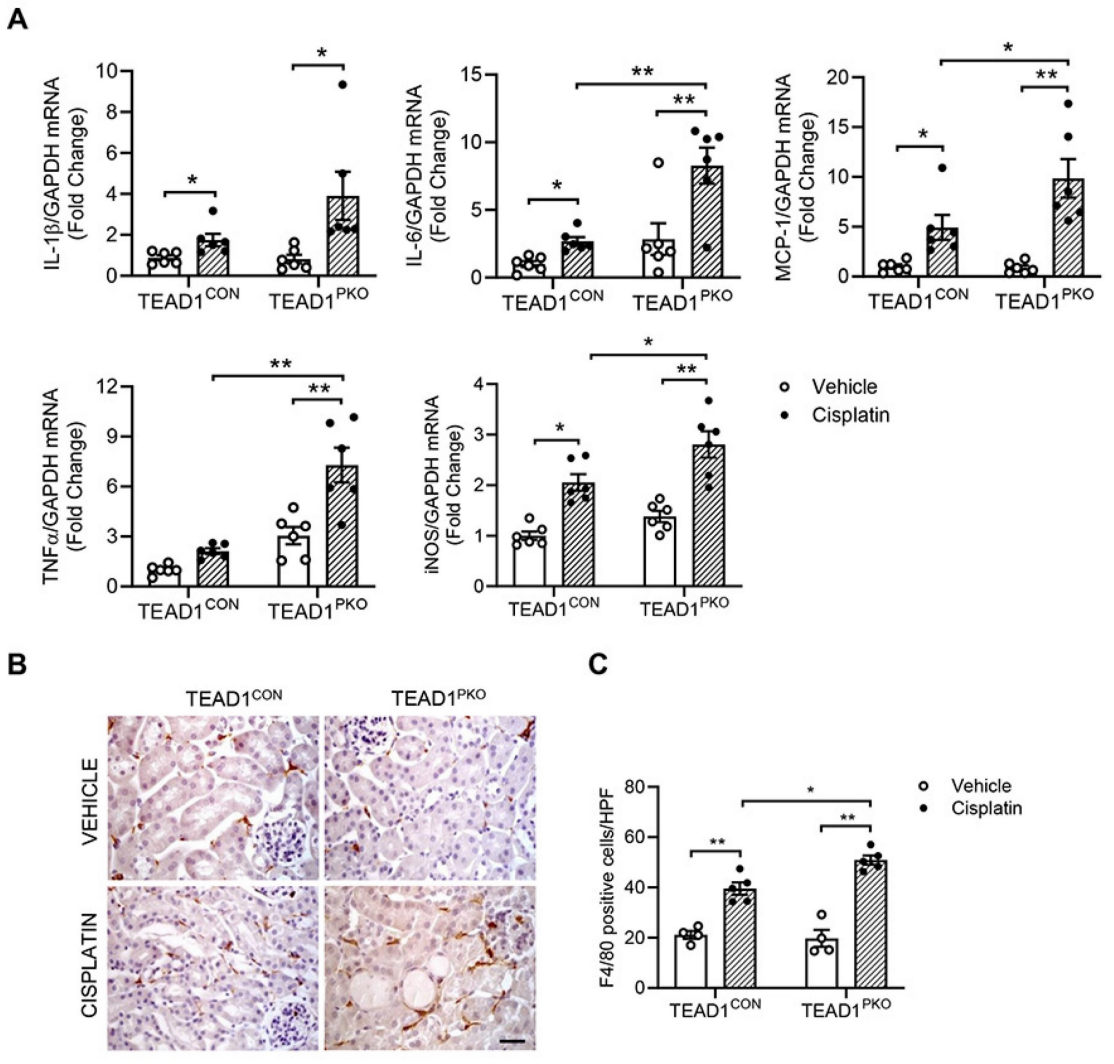
** TEAD1 deficiency upregulates inflammatory response in cisplatin-induced AKI.** (A) Quantitative analysis of IL-1β, IL-6, MCP-1, TNFα and iNOS mRNA expression in kidneys of TEAD1^CON^ and TEAD1^PKO^ mice at 72 h after cisplatin or saline administration. * *P* < 0.05 and ** *P* < 0.01, n = 6 per group (B) Representative photomicrographs of kidney sections immunostained for F4/80 (brown) and counterstained with hematoxylin (blue) in TEAD1^CON^ and TEAD1^PKO^ mice at 72 h after cisplatin or saline administration (C) Quantitative analysis of F4/80 positive cells in kidneys of mice at 72 h after cisplatin or saline administration. * *P* < 0.05 and ** *P* < 0.01, n = 5 per group.

To determine whether TEAD1 contributes to the inflammatory response in cisplatin-induced AKI, we measured pro-inflammatory cytokines in kidneys of mice treated with vehicle or cisplatin. Quantitative real-time PCR showed mRNA levels of IL-1β, IL-6, MCP-1, TNFα, and iNOS were increased in kidneys of TEAD1^CON^ mice treated with cisplatin and even further upregulated in cisplatin-treated TEAD1^PKO^ mice (Figure 4A). In addition, F4/80 immunostaining showed that the infiltration of macrophages was evident in mice with cisplatin treatment, which was augmented in TEAD1^PKO^ mice (Figure 4B-C). Overall, our results demonstrate that genetic deletion of TEAD1 in mice augments cisplatin-induced inflammatory response of the kidneys.

### Knockdown of TEAD1 impairs mitochondrial morphology and induces ROS production

**Figure 5:**
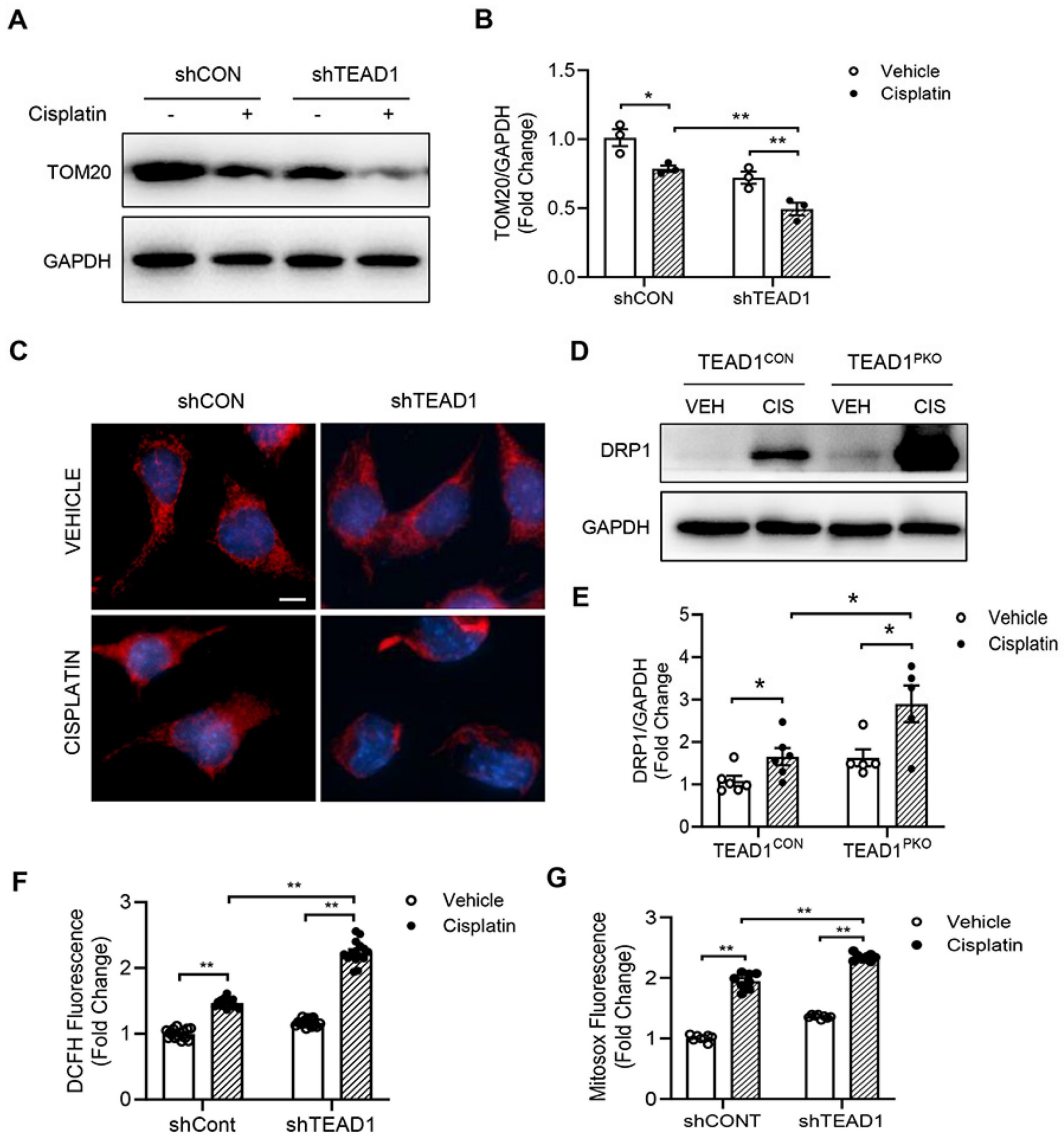
** Knockdown of TEAD1 impairs mitochondrial morphology and induces ROS production.** (A) Representative Western blots show TOM20 protein expression in shCON and shTEAD1 TCMK-1 cells after 24 h of 10 µM cisplatin or vehicle treatment (B) Quantitative analysis of TOM20 protein expression in TCMK-1 cells after 24 h of 10 µM cisplatin or vehicle treatment. * *P* < 0.05 and ** *P* < 0.01, n = 3 per group (C) Representative photomicrographs of TCMK-1 cells with shCON or shTEAD1 and labelled with the mitochondrial dye, MitoTracker Red. Scale bar: 50 μM (D) Representative Western blots show DRP1 protein expression in kidneys of TEAD1^CON^ and TEAD1^PKO^ mice at 72 h after cisplatin or saline administration (E) Quantitative analysis of DRP1 protein expression in mice at 72 h after cisplatin or saline administration. * *P* < 0.05, n = 6 per group (F-G) Detection of intracellular ROS. * *P* < 0.05 and ** *P* < 0.01, n = 8-10 per group.

To determine the mechanism by which TEAD1 regulates cisplatin-induced inflammation and necroptosis, we focused on the mitochondria as previous studies have shown that mitochondrial damage mediates cisplatin-induced AKI. Firstly, we used the mitochondria marker, TOM20, and checked protein expression in TCMK-1 cells transduced with shRNA for Control (shCON) or TEAD1 (shTEAD1). Cisplatin reduced TOM20 expression which was further downregulated with TEAD1 knockdown in TCMK-1 cells (Figure 5A-B). Next, mitochondrial morphology was examined in cisplatin and vehicle-treated TCMK-1 cells labelled with MitoTracker Red (Figure 5C). Mitochondria of vehicle-treated cells were filamentous and showed an elongated and threadlike tubular structure while mitochondria in cisplatin-treated control cells were fragmented and appeared spotted (Figure 5C). Meanwhile, following cisplatin treatment, knockdown of TEAD1 showed more shortened and fragmented cells compared to shCON cells (Figure 5C). The morphology of mitochondria is determined by a balance between fission and fusion 32. Treatment with cisplatin led to upregulation of the mitochondrial fission protein, DRP1, which was exacerbated by TEAD1 deficiency (Figure 5D-E). Oxidative stress is a major response of cisplatin administration; therefore, we examined mitochondrial ROS production using fluorescence probes, MitoSOX Red Mitochondrial Superoxide Indicator and DCFH-DA, in vehicle and cisplatin-treated cells. Cells treated with cisplatin showed increased ROS production, as demonstrated by MitoSOX Red and DCFH-DA probe, and ROS production was higher in cisplatin-treated cells with TEAD1 knockdown compared to cisplatin-treated controls (Figure 5F-G). Taken together, TEAD1 may prevent cisplatin-induced tubular necroptotic cell death via maintaining mitochondrial homeostasis in cisplatin-induced AKI.

### Knockdown of TEAD1 promotes mitochondrial dysfunction

**Figure 6:**
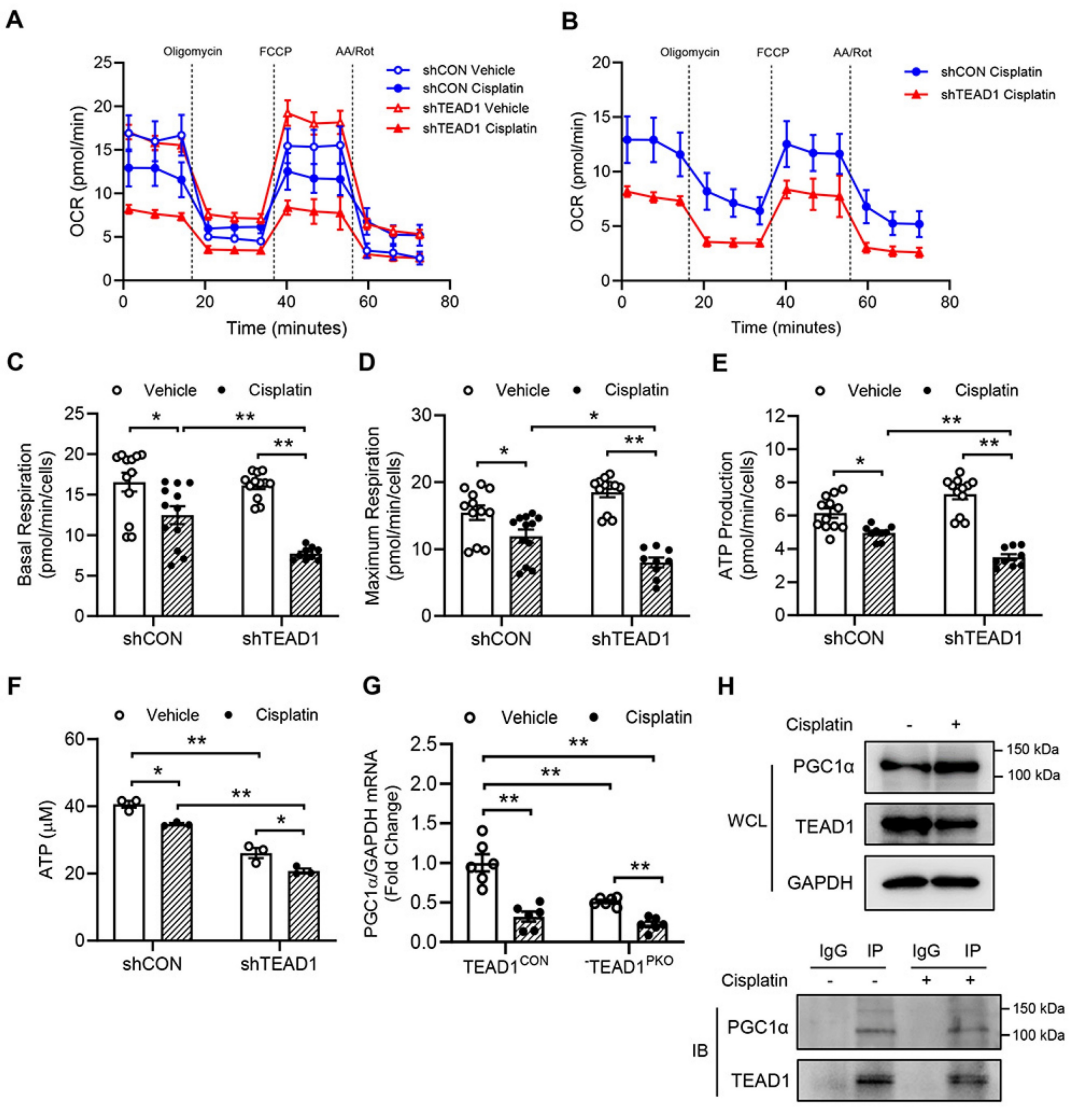
**Knockdown of TEAD1 impairs mitochondrial function.** (A) Seahorse XFe96 analysis of mitochondrial oxygen consumption rate (OCR) in TCMK-1 cells transduced with shCON or shTEAD1 and treated with 10 μM cisplatin or vehicle for 24 h (n = 9-12 cultures per treatment group) (B) Seahorse XFe96 analysis of OCR in TCMK-1 cells transduced with shCON or shTEAD1 and treated with 10 μM cisplatin for 24 h (n = 9-12 cultures per group) (C) Basal respiration rate (D) Maximal respiration rate and (E) ATP production were analysed from OCR. * *P* < 0.05 and ** *P* < 0.01, n = 8 - 12 per group (F) Measurement of ATP production in TCMK-1 cells transduced with shCON or shTEAD1 and treated with 10 μM cisplatin or vehicle for 24 h. * *P* < 0.05 and ** *P* < 0.01, n = 3 per group (G) Quantitative analysis of PGC1α mRNA expression in kidneys of TEAD1^CON^ and TEAD1^PKO^ mice at 72 h after cisplatin or saline administration. ** *P* < 0.01, n = 6 per group (H) Co-immunoprecipitation assay show TEAD1 interacts with endogenous PGC1α in TCMK-1 cells treated with 10 µM cisplatin or vehicle for 24 h. Cell lysates were immunoprecipitated with anti-TEAD1 antibody or control IgG antibody.

To gain further insight into the role of the mitochondria in mediating cisplatin-induced nephrotoxicity, mitochondrial bioenergetics were evaluated in shCON and shTEAD1 cells treated with cisplatin (Figure 6A-B). In shCON cells, cisplatin treatment significantly reduced mitochondrial respiration at basal and maximal levels (Figure 6C-D), with a subsequent decrease in ATP production (Figure 6E), which was further diminished in cisplatin-treated cells with TEAD1 knockdown (Figure 6A-E). To confirm that knockdown of TEAD1 showed reduced ATP levels in response to cisplatin administration, cells were treated with 10 µM cisplatin for 24 h and then ATP levels were measured. The results showed that knockdown of TEAD1 demonstrate reduced ATP production *in vitro* (Figure 6F). PGC1α is recognized as the master regulator of mitochondrial biogenesis and plays a key role in mitochondrial metabolism 33-35. Accordingly, cisplatin administration reduced mRNA levels of PGC1α in both TEAD1^CON^ and TEAD1^PKO^ mice which was further downregulated in TEAD1^PKO^ mice (Figure 6G). TEAD1 is a transcription factor that exerts its transcriptional effects by binding to its co-factor. To determine the association between endogenous TEAD1 and PGC1α, a co-immunoprecipitation assay was performed in TCMK-1 cells treated with cisplatin or vehicle. TCMK-1 cells were immunoprecipitated with TEAD1 or control IgG antibody and then immunoblotted for PGC1α. The results demonstrate that PGC1α could be detected in anti-TEAD1 immunoprecipitates (Figure 6H), indicating that TEAD1 may interact with PGC1α. Taken together, our results demonstrate that deletion of TEAD1 aggravates cisplatin-induced mitochondrial dysfunction through downregulation of PGC1α expression.

## Discussion

Cisplatin is widely used for treatment of various solid tumors, but nephrotoxicity, a major side effect of cisplatin administration, limits the use of cisplatin in cancer therapy. Therefore, understanding the molecular mechanisms of cisplatin-induced AKI may lead to development of novel therapeutic interventions. TEAD1, a major downstream effector of the Hippo signaling pathway, has been reported to regulate cellular proliferation and cell death in a range of cells. However, its role in cisplatin-induced AKI has not yet been established. In this study, TEAD1 expression levels were significantly upregulated in mouse kidneys following cisplatin-induced AKI. Deletion of TEAD1 in murine proximal tubular epithelial cells further promoted renal dysfunction, tubular injury, necroptosis, and inflammation in response to cisplatin. *In vitro* studies demonstrated that knockdown of TEAD1 in tubular epithelial cells following cisplatin treatment increased oxidative stress, reduced ATP production, and impaired mitochondrial bioenergetics. Mechanistically, TEAD1 interacts with peroxisomal proliferator-γ coactivator-1α (PGC-1α), a key regulator of mitochondrial biogenesis, to promote mitochondrial function. Taken together, our study provides evidence for TEAD1 as a novel target in cisplatin-induced AKI.

Numerous studies have extensively investigated apoptosis induced by cisplatin in renal tubular cells 36, 37. Only recently, however, has necroptosis emerged as a focal point of research. Necroptosis is a type of regulated programmed cell death that occurs independently of caspases and plays a role in many pathological processes. In contrast to other cell death mechanisms, regulated necrosis is characterized by an early breakdown of plasma membrane integrity and the release of damage-associated molecular patterns (DAMPs) that promote an inflammatory response leading to significant kidney damage. Necroptosis is characterized by the oligomerization and phosphorylation of receptor-interacting serine/threonine kinase-1 (RIPK1) and RIPK3 as well as the recruitment and phosphorylation of the pseudokinase mixed-lineage kinase domain-like protein (MLKL) upon necrosome formation. Phosphorylation of MLKL promotes its translocation to the plasma membrane where it executes necroptosis by inducing ion influx and rupture of the cell membrane 30, 38, 39. The necroptotic signaling pathway can be activated by several stimuli, including death receptors, IFNs, TLRs, RNA or DNA sensors, toxicity, and metabolic stress 38, 39. Additionally, studies have reported that inhibiting RIPK1 40 with pharmacological agents or deleting RIPK3 or MLKL in mice can mitigate kidney damage and alleviate renal dysfunction 30, 41.

In our study, deletion of TEAD1 in proximal tubular epithelial cells of mice aggravated cisplatin-induced necroptosis as demonstrated by increased RIPK1, RIPK3, and MLKL phosphorylation which triggered an inflammatory response as shown by elevated IL-6, MCP-1, TNFα, and iNOS expression levels. Moreover, mouse tubular epithelial cells treated with cisplatin demonstrated increased RIPK1 and MLKL phosphorylation, which was significantly upregulated in cisplatin-treated cells with TEAD1 knockdown. Our results are consistent with other studies that have reported activation of the necroptotic pathway in mice with cardiomyocyte deletion of TEAD1 20, 21. These studies showed that deletion of *TEAD1* in cardiomyocytes of adult mice induced inflammation and activated the necroptotic pathway, evident by increased RIP1, RIP3, and MLKL expression, leading to extensive cardiomyocyte necroptosis without concurrent apoptosis. Meanwhile, administration of a necroptosis inhibitor, necrostatin-1, effectively rescued heart failure induced by TEAD1 deletion 21. However, the underlying mechanism by which *TEAD1* deficiency promotes necroptosis has not fully been elucidated.

Within the cell, cisplatin undergoes conversion into a highly reactive form, swiftly engaging with thiol-containing antioxidant molecules like glutathione. As a result, the depletion of glutathione triggers heightened oxidative stress within the cells. Furthermore, cisplatin can induce mitochondrial dysfunction and elevate the production of ROS by disrupting the respiratory chain. Mitochondria are the powerhouse of the cell and play a vital role in maintaining cellular homeostasis. Proximal tubular cells are highly enriched with mitochondria, which is required to meet the high energy consumption demands of tubular reabsorption and secretion. However, this mitochondrial enrichment makes these cells vulnerable to various insults such as hypoxia and toxins. Furthermore, substantial research underscores mitochondria as crucial target organelles in AKI 42-48, highlighting mitochondrial dysfunction as a significant aspect in the onset and progression of AKI. Specifically, cisplatin has been found to induce miR-709 upregulation, a microRNA implicated in inhibiting mitochondrial transcription factors like mitochondrial transcription factor A (TFAM)^44^. Moreover, disrupting the genetic regulation of mitochondrial biogenesis transcription factors, such as estrogen receptor alpha (ERα), results in decreased transcription of mitochondrial genes and leads to compromised mitochondrial metabolism characterized by reduced oxygen consumption rates and increased superoxide production, ultimately promoting apoptosis 49. Beyond mitochondrial biogenesis and function, maintaining mitochondrial dynamics, including fusion and fission processes, is imperative to facilitate the creation of new mitochondria and the removal of damaged mitochondria to meet energetic demands 28, 32, 50, highlighting mitochondrial dysfunction as a significant aspect in the onset and progression of AKI.

PGC-1α is a key regulator of mitochondrial biogenesis which has been shown to be involved in AKI 51-54. Moreover, PGC-1α deficiency in the proximal tubule was shown to worsen tubular injury and renal dysfunction in cisplatin-induced AKI 52, as well as the development of diabetic kidney disease and renal fibrosis 35, 55. In our study, PGC1α mRNA expression was significantly downregulated in TEAD1^PKO^ mice exposed to cisplatin nephrotoxicity. This is in line with previous studies demonstrating a role for YAP-TEAD1 signaling in controlling mitochondrial biogenesis 22, 23, 56. Mammoto *et al.* reported that knockdown of TEAD1 using siRNA not only reduced the expression of PGC1α but also suppressed mitochondrial biogenesis, glycolysis, and oxygen consumption rate in endothelial cells 23. Similar observations were also reported in our study where knockdown of TEAD1 in tubular epithelial cells treated with cisplatin significantly induced mitochondrial superoxide levels and ROS production leading to diminished mitochondrial staining intensity. These observations correlated with suppressed mitochondrial metabolism, evident in reduced basal and maximal respiration, lower total mitochondrial respiration, and downregulated ATP production. Moreover, our study revealed that TEAD1 interacts with PGC1α to preserve mitochondrial function. In a separate study, TEAD1 was shown to play a role in the reprogramming of cardiac fibroblasts into induced cardiomyocytes, by regulating mitochondrial biogenesis via expression of PGC-1 17. Additional studies, however, are warranted to determine how TEAD1 regulates PGC1α and mitochondrial function.

Mitochondrial dysfunction constitutes an important aspect of kidney etiopathogenesis and progression 45, 50. However, the molecular mechanisms are still largely unknown. Growing lines of evidence indicate that Hippo-TEAD1 pathway plays a role in cellular bioenergetics. Previously, genome-wide transcriptome and ChIP-seq analysis unveiled that TEAD1 directly activates a significant array of nuclear DNA-encoded mitochondrial genes essential for electron transfer complex assembly and ATP production 21. Furthermore, evaluation of mitochondrial bioenergetics in isolated mitochondria of adult hearts revealed a substantial decrease in respiratory rates upon TEAD1 loss, which was associated with diminished activity and expression of electron transport chain complex I. Additionally, deletion of TEAD1 in primary cardiomyocytes further confirmed reduced aerobic respiration and maximal mitochondrial oxygen consumption capacity, increased mitochondrial ROS, disruption of the structure of mitochondria, and reduced complex I-IV driven oxygen consumption and ATP levels 19-21. Another study also demonstrated that activation of Hippo signaling mediates mitochondrial damage by repressing mitochondrial genes, which promotes the development of dilated cardiomyopathy 56. Collectively, these findings reveal TEAD1 as a critical transcriptional regulator governing a vast network of genes essential for mitochondrial integrity and function. Our* in vitro* studies following TEAD1 knockdown in tubular epithelial cells have showed that TEAD1 deletion promoted necroptosis and impaired mitochondrial function, which support an important role of TEAD1 in the regulation of cisplatin-induced kidney injury.

In addition to the role of PGC1α in AKI, several studies have highlighted the involvement of GSK3β in AKI 57, 58. GSK3β, initially known for regulating glycogen metabolism, plays a key role in various cellular processes such as cell death, cytoskeleton organization, and insulin signaling 59. Specifically, GSK3β is implicated in oxidative stress-induced mitochondrial dysfunction and necroptotic cell death in renal tubular cells, induced by the herbicide paraquat 60. Moreover, its activity is influenced by ROS, and inhibiting GSK3β with selective inhibitors like TDZD-8 or lithium has been shown to reduce necroptosis, apoptosis, and kidney dysfunction 60. One study showed that overexpression of TEAD1 in skeletal muscle activates GSK3β and decreases nuclear levels of β-catenin and NFATc1/c3, which was reversed by mechanical overload 61 suggesting a potential link between TEAD1 and GSK3β in regulating AKI, however further studies are required to confirm this connection.

In summary, our study demonstrates for the first time that genetic deletion of TEAD1 in proximal tubular epithelial cells of kidneys aggravated necroptosis and inflammation, resulting in mitochondrial dysfunction and kidney injury. Therefore, TEAD1 may represent a novel therapeutic target in cisplatin-induced AKI.

## Supplementary Material

Supplementary figures and table.
