# Supplementary material for: TEAD1 Prevents Necroptosis and Inflammation in Cisplatin-Induced Acute Kidney Injury Through Maintaining Mitochondrial Function
Source: Int J Biol Sci. 2025 Jan 1;21(2):565–78. doi: 10.7150/ijbs.104335 (PMC11705647; doi:10.7150/ijbs.104335)
Supplement: Supplementary file 1 — Supplementary figures and table. [file ijbsv21p0565s1.pdf]

SUPPLEMENTAL MATERIAL

TEAD1 Prevents Necroptosis And Inflammation In Cisplatin-Induced Acute Kidney Injury Through Maintaining Mitochondrial Function

Melanie Tran[1], Baihai Jiao[1], Hao Du[1], Dong Zhou[1], Vijay Yechoor[3], Yanlin Wang[1,2,4]

[1]Division of Nephrology, Department of Medicine, University of Connecticut School of Medicine, Farmington, CT, USA; [2] Department of Cell Biology, University of Connecticut School of Medicine, Farmington, CT, USA; [3]Department of Medicine, University of Pittsburgh, Pittsburgh, PA, USA; [4]Renal Section, Veterans Affairs Connecticut Healthcare System, West Haven, CT, USA.

* Correspondence should be addressed to:

Yanlin Wang, MD, PhD

Division of Nephrology, Department of Medicine

University of Connecticut School of Medicine

263 Farmington Avenue, Farmington, CT 06030-1405

E-mail: yanlwang@uchc.edu

Supplementary Figure 1. Generation of proximal-tubule specific TEAD1 knockout mice (TEAD1^{PKO}). (a) Schematic representation of genetic crosses to conditionally delete TEAD1 in proximal tubules of kidneys (b) Representative photomicrographs of kidney sections immunostained for TEAD1 (brown) and counterstained with hematoxylin (blue) in TEAD1^{CON} and TEAD1^{PKO} mice. Proximal tubules are indicated by black arrows. Scale bar: 50 µM.

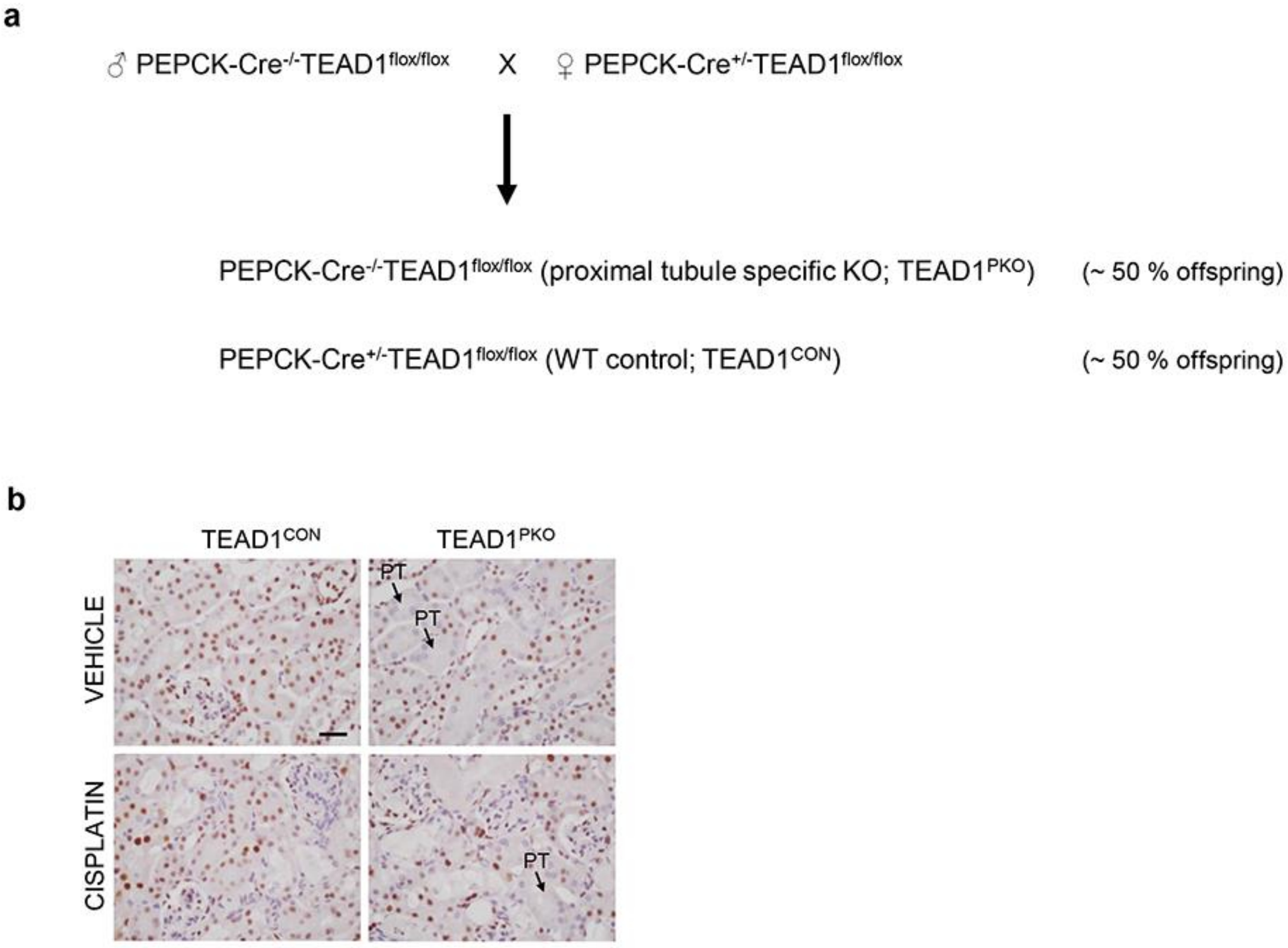

Supplementary Figure 2. Knockdown of TEAD1 in TCMK-1 cells promote necroptosis. (a) Representative Western blots show phosphorylated RIP1, RIP1, phosphorylated RIP3, RIP3, phosphorylated MLKL and MLKL protein expression in TCMK-1 cells transduced with shCON or shTEAD1 and treated with 10 µM cisplatin or vehicle for 24 h (b) Quantitative analysis of phosphorylated RIP1, phosphorylated RIP3, and phosphorylated MLKL protein expression in TCMK-1 cells treated with 10 µM cisplatin or vehicle for 24 h. * $P < 0.05$ and ** $P < 0.01$, n = 3 per group.

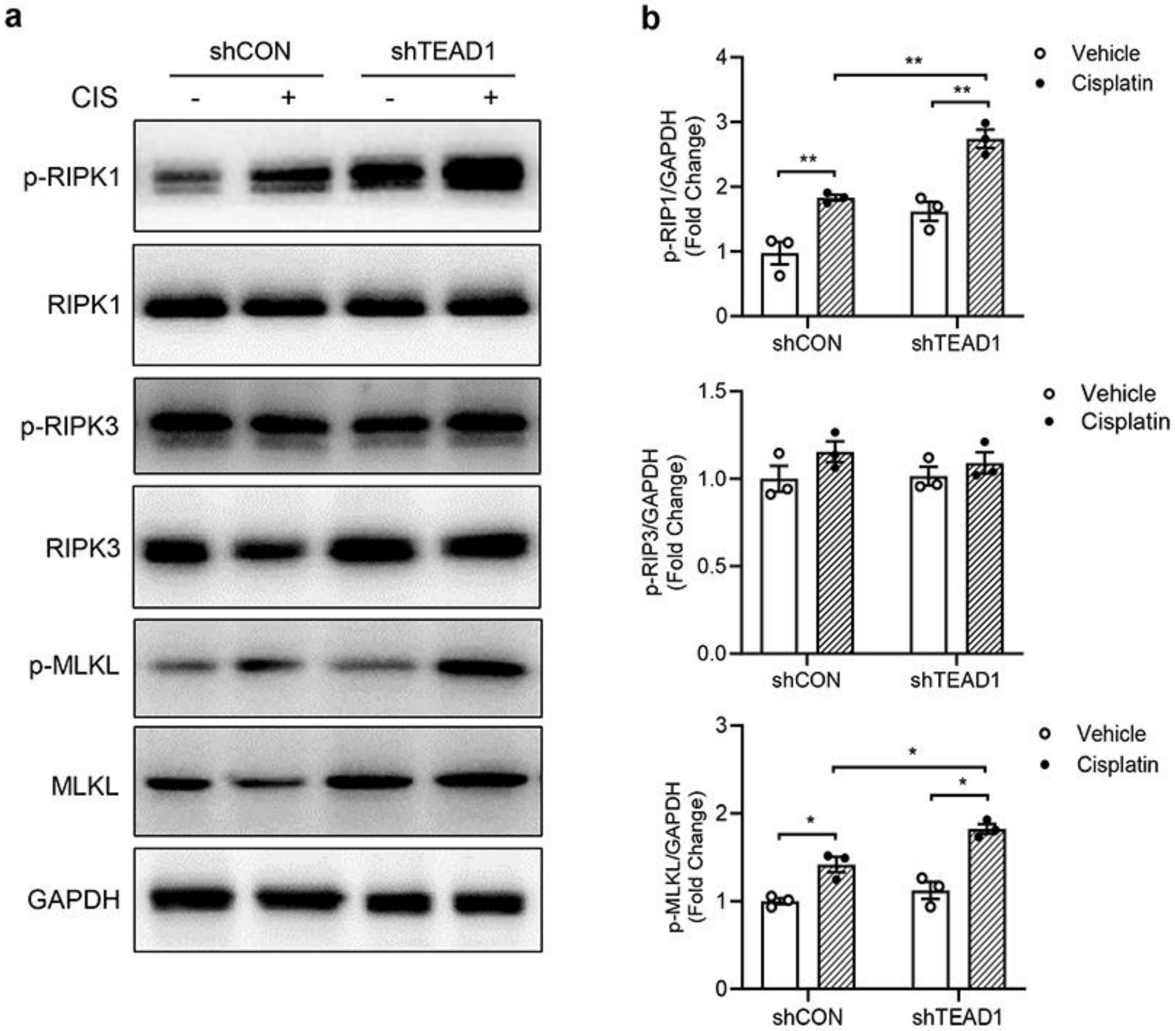

Supplementary Table 1. Sequence of primers used for genotyping and real time RT-PCR.

Primer	Forward primer	Reverse primer
TEAD1 genotyping	GCCTTCTGAGTGCTAGCATTAAAGG	AAGGCAGACTCCTTCATTGGATGG
PEPCKCre genotyping	ACCTGAAGATGTTCGCGATTATCT	ACCGTCAGTACGTGAGATATCTT
IL-1β	CTTCAGGCAGGCAGTATCACTCAT	TCTAATGGGAACGTCACACACCAG
IL-6	AGGATACCACTCCCAACAGACCTG	CTGCAAGTGCATCATCGTTGTTCA
MCP-1	TCACCTGCTGCTACTCATTCACCA	TACAGCTTCTTTGGGACACCTGCT
TNFα	CATGAGCACAGAAAGCATGATCCG	AAGCAGGAATGAGAAGAGGCTGAG
iNOS	GGCAGCCTGTGAGACCTTTG	GCATTGGAAGTGAAGCGTTTC
MLKL	ACCCTTCAGAGGCACAACAC	TGTCATTGGATTCGGTGGGG
RIP3	GAAGACACGGCACTCCTTGGTA	CTTGAGGCAGTAGTTCTTGGTGG
PGC1α	AAACTTGCTAGCGGTCCTCA	TGGCTGGTGCCAGTAAGAG
GAPDH	CCAATGTGTCCGTCGCGTGGATCT	GTTGAAGTCGCAGGAGACAACC
